# TightRope Technique as an Alternative Surgical Approach for Acromioclavicular Joint Dislocation: A Case Report of a Minimally Invasive Fixation and Review of Literature

**DOI:** 10.7759/cureus.83875

**Published:** 2025-05-11

**Authors:** Arın Celayir, Erdem Sahin, Huseyin Botanlioglu

**Affiliations:** 1 Department of Orthopedics and Traumatology, Cerrahpaşa Faculty of Medicine, Istanbul University-Cerrahpaşa, Istanbul, TUR

**Keywords:** acromioclavicular joint dislocation, coracoclavicular stabilization, minimally invasive fixation, shoulder surgery, tightrope technique

## Abstract

Acromioclavicular (AC) joint dislocations are common shoulder injuries, particularly in athletes and individuals exposed to high-impact trauma. The management of these injuries varies depending on severity, with the Rockwood classification guiding treatment decisions. While hook plates, coracoclavicular (CC) ligament reconstructions, and screw fixations remain the standard surgical techniques, a minimally invasive alternative such as the TightRope technique is emerging as an effective option. This report presents a case of a 34-year-old male with a Rockwood Type III AC joint dislocation, successfully treated with TightRope fixation. Postoperative radiographic evaluations confirmed optimal implant positioning and stable fixation, with the patient achieving full range of motion by the second postoperative month. The TightRope technique provides dynamic joint stabilization, early mobilization, and fewer implant-related complications compared to traditional methods. Given its advantages, it should always be considered as an alternative treatment option, particularly for young and athletic individuals who require early functional recovery. Expanding awareness and further clinical research may help establish its broader use in surgical practice.

## Introduction

Acromioclavicular (AC) joint injuries are common shoulder trauma, frequently seen in athletes, manual workers, and individuals exposed to high-impact activities. These injuries often result from direct trauma to the lateral aspect of the acromion, leading to varying degrees of ligamentous damage and clavicular displacement [1]. The Rockwood classification system is widely used to categorize AC joint dislocations into six types, ranging from mild sprains (Types I and II) to complete ligament ruptures with severe displacement (Types IV-VI). While Type I and II injuries are typically managed conservatively with rest, immobilization, and rehabilitation, higher-grade dislocations (Types IV-VI) often necessitate surgical intervention to restore shoulder function and stability [2].

Several surgical techniques have been developed to address AC joint dislocations, each with its advantages and limitations. Hook plate fixation is a well-established method that provides rigid stabilization, but it frequently requires a second surgery for implant removal and may cause subacromial impingement or erosion. Coracoclavicular (CC) ligament reconstruction with autografts or allografts offers a biological solution by restoring ligament continuity, yet it involves longer surgical times and potential donor site morbidity. Screw fixation can also be used for CC stabilization, but implant failure, screw migration, and loss of reduction are common concerns [3].

In contrast, the TightRope technique represents a less invasive alternative that has gained attention for its dynamic stabilization and lower complication rates [4]. Unlike rigid fixation methods, TightRope allows for controlled micromovement, promoting biological healing of the AC and CC ligaments. Additionally, it eliminates the need for hardware removal, reduces the risk of subacromial irritation, and supports faster postoperative recovery. Nevertheless, its widespread adoption remains limited.

While hook plates, CC ligament reconstructions, and screw fixations remain the mainstay of surgical treatment, the TightRope technique provides a promising alternative with minimally invasive benefits and dynamic joint stability. In our patient, we successfully applied the TightRope technique, demonstrating its efficacy as an alternative approach that should be considered as a viable option. This technique is particularly advantageous in young and athletic patients, where preserving shoulder mobility and early return to activity are crucial. Expanding awareness and further clinical research may help establish TightRope fixation as a more common and preferred surgical option for appropriately selected cases.

## Case presentation

A 34-year-old male patient presented to our emergency department following a fall, complaining of widespread right shoulder pain. Informed consent was obtained before any procedure was performed. The patient has a known history of hepatitis B carrier status but no other comorbidities and does not take any regular medication. He reported experiencing an AC joint dislocation on the same side one year ago, which was treated conservatively with a shoulder sling.

**Figure 1:**
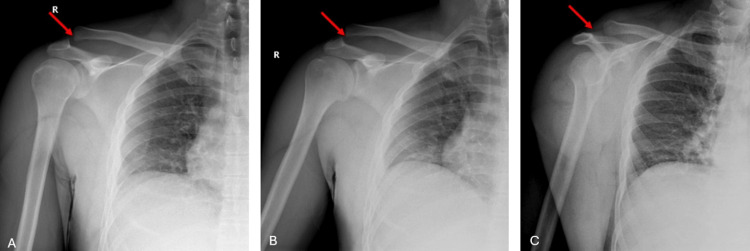
Initial shoulder radiographs of the patient (A) AP view. (B) True AP view. (C) Axial rotation view. All images demonstrate an AC joint dislocation. The red arrows indicate the AC joint separation. AC, acromioclavicular

On physical examination, asymmetry of the right shoulder was observed. There was tenderness at the right AC joint level upon palpation, and the right shoulder appeared more elevated compared to the left. Radiological evaluation was performed, revealing a right AC joint dislocation (Figure 1).

**Figure 2:**
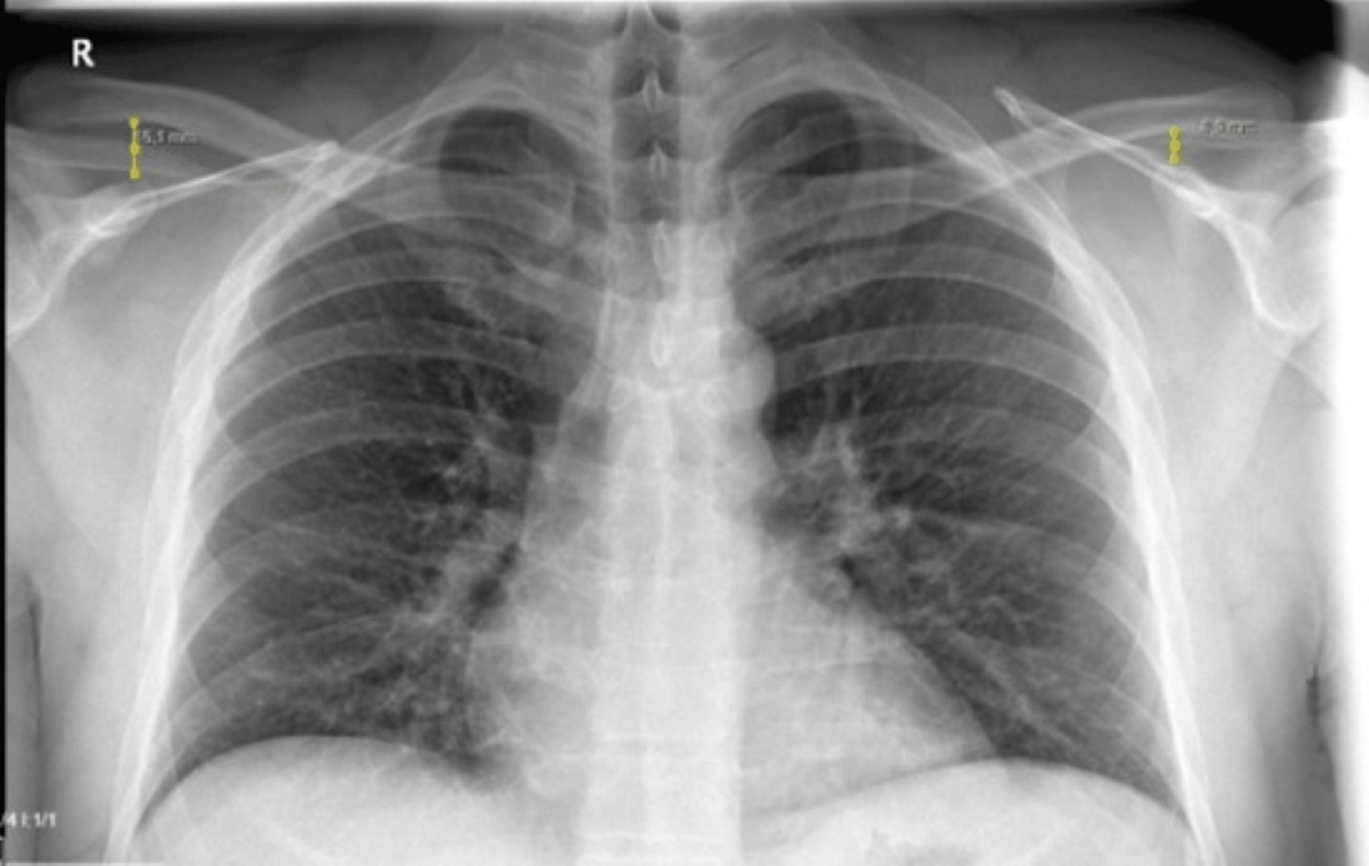
PA shoulder radiograph of the patient The image shows bilateral CC distance measurements: 16 mm on the right side and 8 mm on the left. The red arrows indicate the AC joint separation. AC, acromioclavicular; CC, coracoclavicular

The chest X-ray showed the CC distance measured at 16 mm on the right side and 8 mm on the left (Figure 2).

The patient’s dominant side is the right, and he works as a laborer. Based on the Rockwood classification, the injury was assessed as a Type III AC joint dislocation, and surgical treatment was planned (Table 1).

**Table 1:** TightRope surgical technique for AC joint fixation TightRope surgical technique for AC joint fixation

Surgical step	Description
Step 1: Incision and exposure	A small skin incision (3-5 cm) is made just above the clavicle, proximal to the AC joint. Soft tissues are carefully dissected to expose the clavicle and coracoid process.
Step 2: Fluoroscopic guidance and drilling	Under C-arm fluoroscopy, a guidewire is inserted through the clavicle and coracoid process. A cannulated drill is then used to create a transosseous tunnel through both structures.
Step 3: Suture button implant placement	The EndoButton/TightRope device, comprising a strong synthetic fiber suture loop attached to a small titanium button, is passed through the drilled tunnel. The implant is then tightened, securing the clavicle to the coracoid while maintaining physiological motion.
Step 4: Ligament reconstruction and reduction confirmation	The AC ligament is reconstructed using additional sutures if necessary. Reduction of the AC joint is confirmed under fluoroscopy.
Step 5: Wound closure and postoperative care	The wound is closed in anatomical layers. Postoperative radiographs are obtained to evaluate the quality of fixation.

Surgery was initiated for the fixation of the right AC joint dislocation using the TightRope technique. During the surgery, the scapula was positioned in an upright orientation. The fluoroscope was also adjusted to visualize the scapula in an AP view. The entry point was planned approximately 2-3 cm in the axial plane and about 5 cm from the lateral border of the acromion in the AP plane. The target was set beneath the coracoid process, aligned to pass through the midpoint of the clavicle.

**Figure 3:**
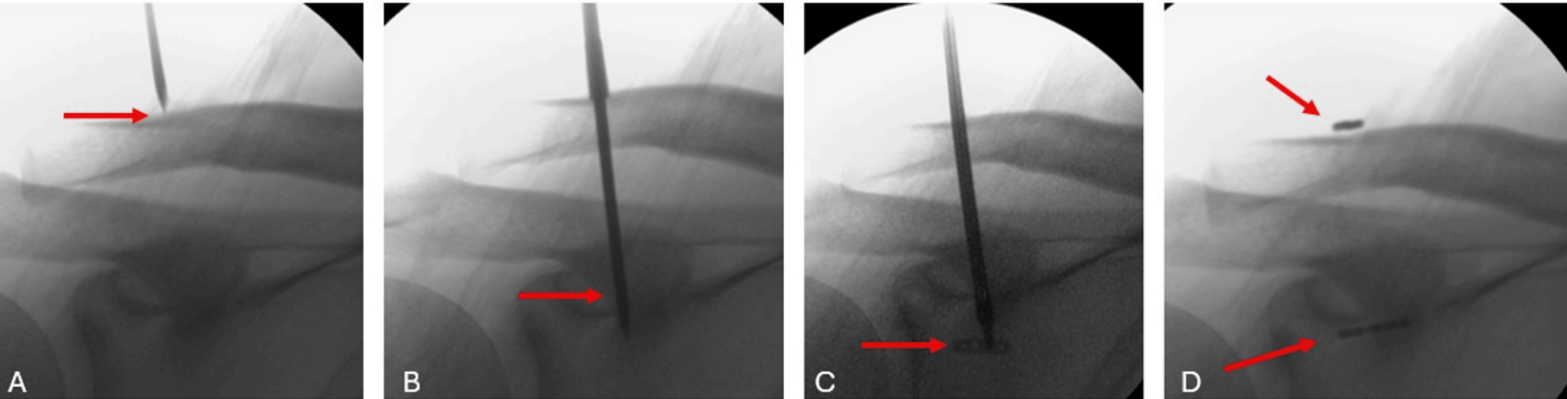
Intraoperative fluoroscopic images of the patient (A-D) Sequential images showing the creation of a 4.5 mm CC tunnel using a Kirschner wire for TightRope placement. The red arrows indicate the tunnel site. CC, coracoclavicular

**Figure 4:**
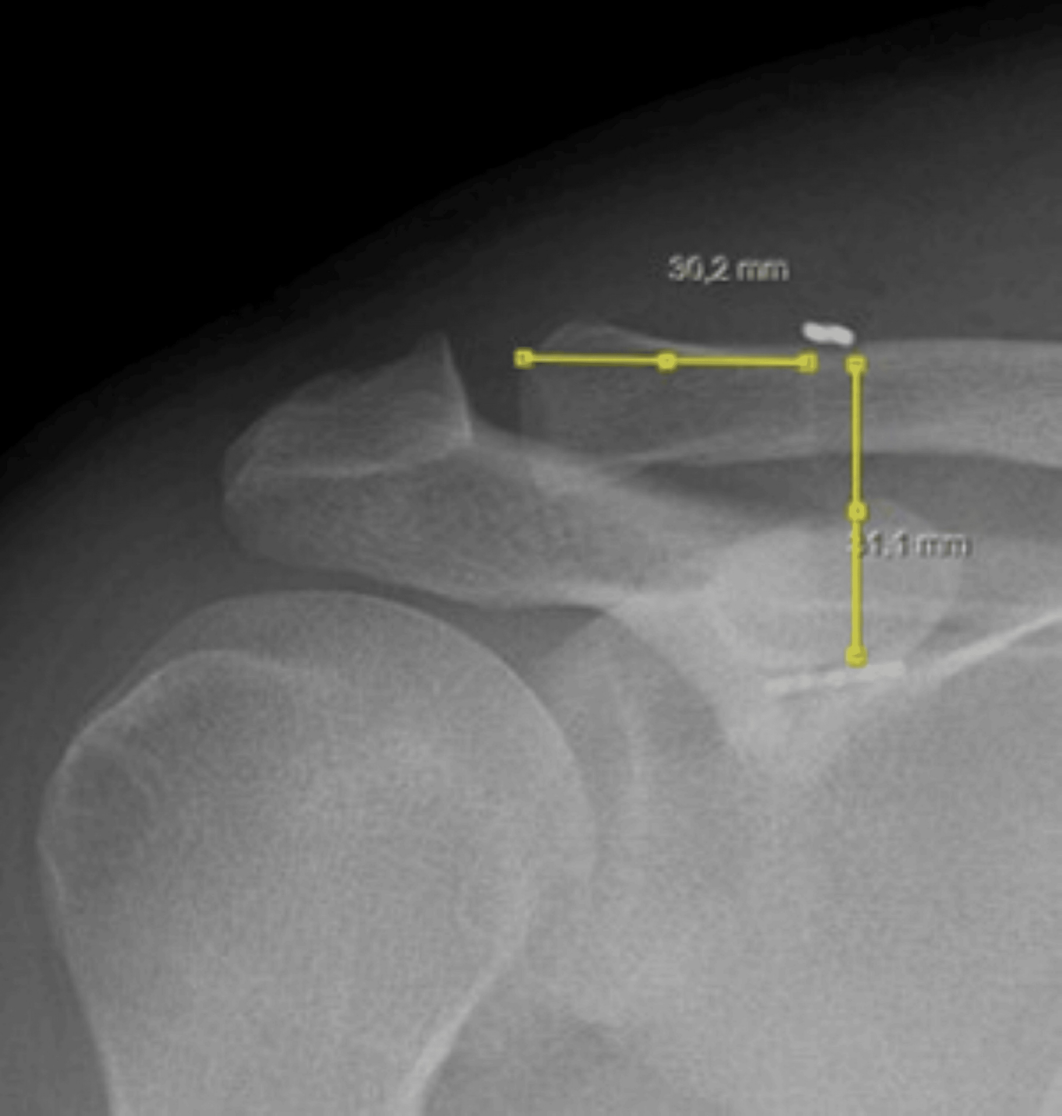
Tunnel entry point approximately 3 cm from the AC joint AC, acromioclavicular

A longitudinal incision of approximately 2 cm was made about 4 cm proximal to the right AC joint. Under fluoroscopic guidance, percutaneous drilling was performed through the clavicle and coracoid process, and a 4.5 mm tunnel was created. Then, using a button retainer (EndoButton), the implant was passed through the drilled holes, and AC ligament reconstruction was performed (Figure 3, Figure 4).

**Figure 5:**
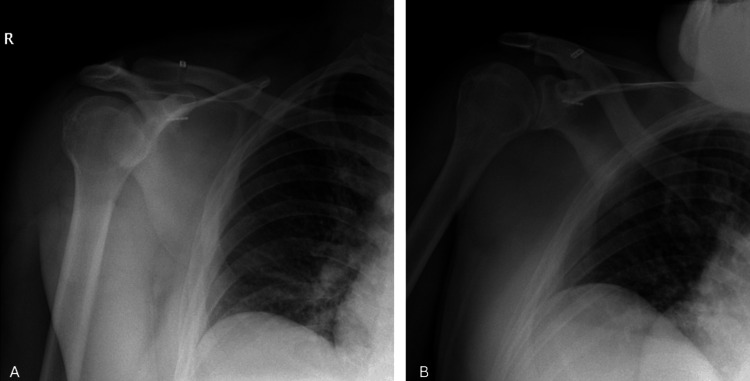
Postoperative radiographic images of the patient (A) AP shoulder radiograph. (B) True AP shoulder radiograph. Both images confirm correct implant placement and adequate fixation.

Reduction was confirmed as appropriate. The skin and subcutaneous tissues were closed in accordance with their anatomical layers. No intraoperative or early postoperative complications were observed. Postoperative x-ray revealed no pathology (Figure 5).

**Figure 6:**
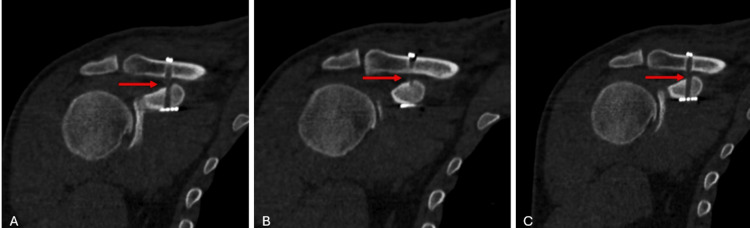
Postoperative coronal CT images of the patient The images show the tunnel placement in the coracoid bone and clavicle, confirming proper implant positioning within the tunnel. The red arrows indicate the tunnel.

**Figure 7:**
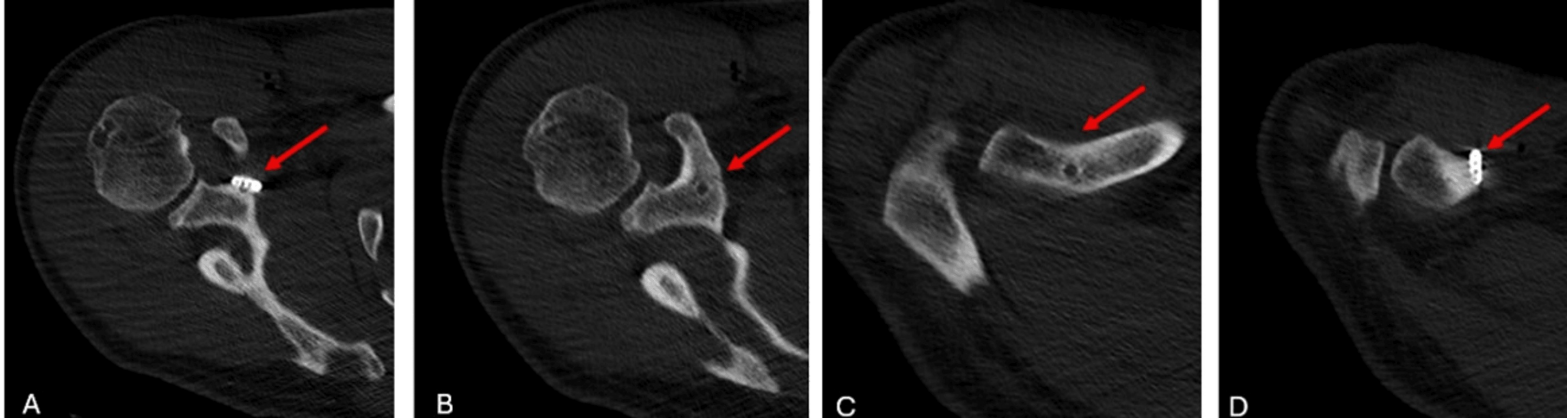
Postoperative axial CT images of the patient The images show the tunnel placement in the coracoid bone and clavicle, confirming proper implant positioning within the tunnel. The red arrows indicate the tunnel.

**Figure 8:**
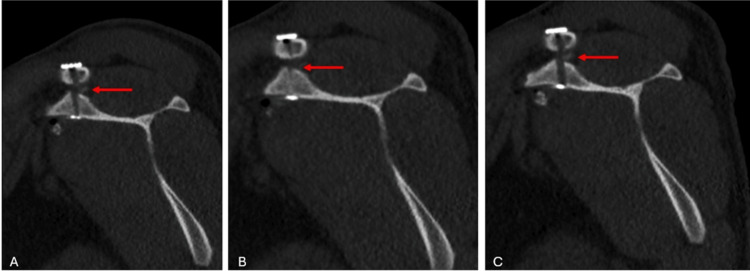
Postoperative sagittal CT images of the patient The images show the tunnel placement in the coracoid bone and clavicle, confirming proper implant positioning within the tunnel. The red arrows indicate the tunnel.

**Figure 9:**
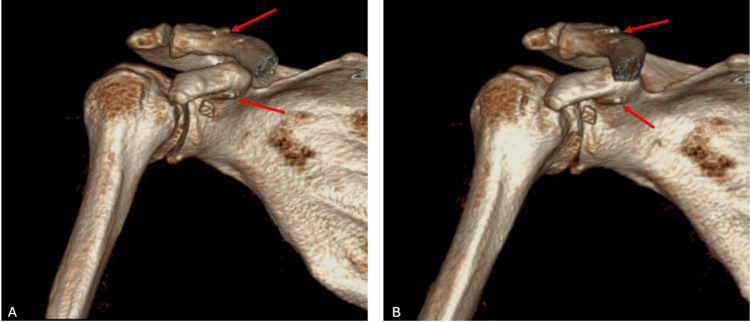
Postoperative 3D reconstructed thin-slice CT images of the patient The images show the tunnel placement in the coracoid bone and clavicle, confirming proper implant positioning within the tunnel. The red arrows indicate the EndoButtons.

The patient was discharged one day after surgery with a shoulder sling. Passive elbow exercises were initiated at the time of discharge. At the two-week postoperative follow-up, the sutures were removed, and the patient was instructed to begin pendulum exercises. A shoulder CT scan was performed during the two-week postoperative follow-up. The postoperative CT scan confirmed that the TightRope materials were within the tunnel and that the fixation was appropriate (Figure 6, Figure 7, Figure 8, Figure 9).

At the two-month postoperative follow-up, the patient had a full range of motion (ROM) and was able to actively use the shoulder without limitations. The surgical wound had healed with minimal to no visible scarring, and both shoulders appeared symmetric in posture and contour.

## Discussion

AC joint dislocations are managed using various surgical techniques, each with its own advantages and limitations. The most commonly used approaches include hook plate fixation, CC ligament reconstruction, and screw fixation, all of which have demonstrated efficacy in stabilizing the joint. However, these techniques present specific challenges, making it necessary to explore alternatives such as the TightRope technique, particularly in certain patient populations.

Hook plate fixation is a widely used method for high-grade AC joint dislocations, providing rigid stabilization by anchoring the clavicle to the acromion [5]. However, implant removal is often required, and complications such as subacromial impingement and acromial erosion can lead to persistent pain and restricted motion [6].

CC ligament reconstruction, using autografts or allografts, restores anatomical stability but involves a more invasive approach. This leads to longer operative times and potential donor site morbidity, with additional concerns regarding graft elongation and improper tensioning, which may compromise long-term joint stability [7].

Screw fixation, such as Bosworth screws, provides strong fixation but carries the risk of screw migration, implant loosening, and breakage. The rigid nature of screw fixation may also increase stress on surrounding structures, potentially affecting joint mobility [8].

The TightRope technique has emerged as a minimally invasive alternative that effectively addresses the shortcomings of traditional fixation methods. This technique has gained popularity, particularly for high-demand patients such as athletes and manual laborers, due to its ability to provide stability without excessive surgical trauma [9]. It involves a smaller incision, resulting in less soft tissue damage and a lower risk of complications. Unlike hook plate fixation, the TightRope technique does not require secondary implant removal, reducing morbidity and eliminating the risk of hardware-related discomfort. Additionally, its dynamic stabilization allows for controlled micromovements, promoting natural ligament healing while maintaining joint flexibility. This leads to faster postoperative recovery, facilitating early mobilization and rehabilitation, which is especially critical for individuals needing an early return to activity.

Recent research has further emphasized the long-term advantages of TightRope fixation over hook plate fixation. A study by Ko et al. (2023) compared long-term clinical outcomes of TightRope versus hook plate fixation for acute AC joint dislocations [10]. Their findings demonstrated that patients treated with the TightRope technique experienced better functional outcomes, lower complication rates, and reduced need for revision surgeries. The study highlighted that hook plates often lead to complications such as subacromial impingement, implant-related pain, and acromial osteolysis, necessitating implant removal in a significant percentage of cases. In contrast, TightRope fixation showed lower rates of implant-related irritation and allowed earlier return to physical activity [11].

Moreover, the study reinforced that TightRope fixation provides greater long-term joint mobility, reduces the risk of postoperative stiffness, and minimizes soft tissue disruption compared to hook plate fixation. Given these advantages, the TightRope technique should be considered a preferred treatment option, particularly in younger, active individuals who require a stable and functional recovery. However, further large-scale studies are needed to optimize surgical indications, refine fixation techniques, and improve patient selection criteria for achieving the best clinical outcomes.

In our case, a 34-year-old male with a Rockwood Type III AC joint dislocation was successfully treated using the TightRope technique. Postoperative CT imaging confirmed proper implant positioning and stable fixation, and the patient achieved full ROM by the second postoperative month. The early functional recovery and absence of implant-related complications further reinforce the advantages of this approach over traditional fixation methods.

Although not yet the gold standard, the TightRope technique offers minimally invasive stabilization, faster rehabilitation, and fewer implant-related complications, making it a strong alternative. Given its advantages, especially for young, active patients, further studies are needed to establish its long-term effectiveness compared to traditional AC joint fixation methods.

## Conclusions

The TightRope technique is a minimally invasive and effective alternative for treating AC joint dislocations, especially in young, athletic, and high-demand patients. Compared to hook plates, CC ligament reconstruction, and screw fixation, it provides dynamic stabilization, early rehabilitation, and fewer implant-related complications. Our case demonstrated successful fixation, rapid recovery, and a full ROM by two months, reinforcing its clinical advantages. While not yet the gold standard, its efficacy warrants further research to establish its role in AC joint stabilization and long-term outcomes.

## References

[1] Babhulkar A, Pawaskar A (2014). Acromioclavicular joint dislocations. Curr Rev Musculoskelet Med.

[2] Bannister GC, Wallace WA, Stableforth PG, Hutson MA (1992). A classification of acute acromioclavicular dislocation: a clinical, radiological and anatomical study. Injury.

[3] Haugaard KB, Bak K, Ryberg D, Muharemovic O, Hölmich P, Barfod KW (2024). Acromioclavicular joint dislocation Rockwood type III and V show no difference in functional outcome and 91% recovered well without the need for surgery. Knee Surg Sports Traumatol Arthrosc.

[4] Chen RE, Gates ST, Vaughan A, Santoro A, Reddy Y, Williams GR, Namdari S (2023). Complications after operative treatment of high-grade acromioclavicular injuries. J Shoulder Elbow Surg.

[5] Shaty W (2024). The results of hook plate fixation in acute acromioclavicular joint dislocation and distal clavicle fractures. Orthop Rev (Pavia).

[6] McKee MD (2016). Operative fixation of chronic acromioclavicular joint dislocation with hook plate and modified ligament transfer. J Orthop Trauma.

[7] Cain EL Jr, Parker D (2023). Open anatomic coracoclavicular ligament reconstruction for acromioclavicular joint injuries. Clin Sports Med.

[8] Tiefenboeck TM, Popp D, Boesmueller S (2017). Acromioclavicular joint dislocation treated with Bosworth screw and additional K-wiring: results after 7.8 years - still an adequate procedure?. BMC Musculoskelet Disord.

[9] Mahmoodian A, Yavari P, Moshkdar P, Karimimatloub S, Eslami S, Boroujeni MS, Mohammadsharifi G (2021). Outcomes of acromioclavicular joint dislocation using tightrope arthroscopy. Int J Burns Trauma.

[10] Ko SH, Lee CC, Jeon YD, Han JW, Lee KJ (2023). Long-term clinical outcomes after TightRope versus hook plate fixation for acute acromioclavicular joint dislocation. Orthop J Sports Med.

[11] Yoon JP, Lee BJ, Nam SJ, Chung SW, Jeong WJ, Min WK, Oh JH (2015). Comparison of results between hook plate fixation and ligament reconstruction for acute unstable acromioclavicular joint dislocation. Clin Orthop Surg.

